# Plateau and Patella: A Framework for Ipsilateral Injury Fixation

**DOI:** 10.7759/cureus.67994

**Published:** 2024-08-28

**Authors:** Sandeep Damaraju, Ahmed Eldesoki, Shafiq A Shahban

**Affiliations:** 1 Trauma and Orthopaedics, Walsall Healthcare NHS Trust, Walsall, GBR

**Keywords:** early mobilisation, knee trauma, open reduction internal fixation, lateral tibial plateau, tibia plateau fracture, patella fracture

## Abstract

Ipsilateral patella and tibial plateau fractures represent an extremely rare injury pattern. They are seldom discussed in literature, with no frameworks for management being reported that we were able to find. We report our experience and management of such an injury, suffered by a 57-year-old female patient with good premorbid functional status, by direct trauma to the right knee. Preoperatively, she was managed in a knee splint to aid elevation and help control her pain. We undertook fixation of both the patella and tibia through a midline incision. Postoperatively, we used a hinged knee brace, initially locked in extension, to allow gradual flexion at two weekly follow-ups. She has suffered no postoperative complications thus far at three months.

We hope to highlight a novel management plan for this rare and complex fracture pattern, for which no prior published management evidence exists. As such, we submit the key principles from which our operative plan was derived to aid in the management of such injuries in the future.

## Introduction

Management of fractures to the patella and/or tibial plateau can present numerous surgical challenges if approached incorrectly, affirming the importance of formulating and executing a well-evidenced and robust management plan for addressing these injuries. Ipsilateral patella and tibial plateau fractures are extremely rare injury patterns, with no complete management plans previously reported in existing literature. We report a case of a 57-year-old female patient who sustained both injuries on the same leg from a direct impact injury during an assault. These injuries were managed initially in a knee splint. A CT scan was then performed to help with surgical planning. For definitive management, we performed a simultaneous open reduction internal fixation of both injuries through a single midline incision. Postoperatively, the patient was placed in a hinged knee brace and flexion was allowed gradually at routine follow-up. She was discharged home with no complications postoperatively and remained complication-free up to a three-month follow-up. As literature searches revealed no previously reported cases of both simultaneous injuries affecting the same leg, we present our management as a framework for managing future cases.

## Case presentation

Presentation and initial management 

A 57-year-old female patient presented to our emergency department after being assaulted. She had significant bruising to her right knee and was unable to straight leg raise or weight-bear on this leg.

She mobilised independently before the injury, and her only medical comorbidity was chronic obstructive pulmonary disease. This was managed with inhalers.

X-rays were undertaken in the emergency department, which demonstrated a right-sided patellar fracture with an ipsilateral tibial plateau fracture.

**Figure 1:**
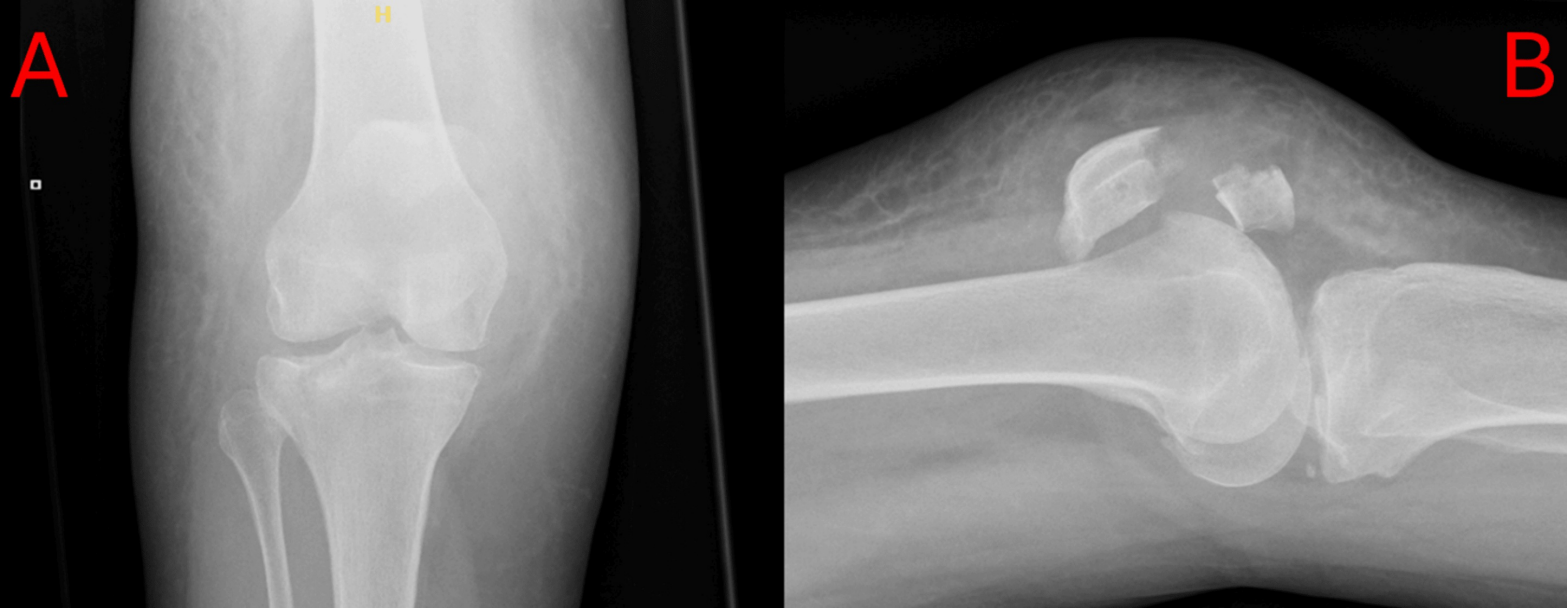
Preoperative anterior to posterior (a) and lateral (b) X-rays of the injury A) Plain anterior to posterior radiograph of the patient's right knee. This image shows a Schatzker 2 [1] lateral condylar fracture; B) Plain lateral radiograph of the patient's right knee. This image shows a displaced, midbody patellar fracture with associated haematoma.

Given the severity of the injury, the patient was placed in a knee splint and admitted to the local Trauma and Orthopaedic service. A computerised tomography (CT) scan was undertaken to plan surgical intervention. This showed a Schatzker 2 [1] comminuted fracture of the right lateral tibial plateau, a depressed lateral condyle fracture involving the joint surface. There was a horizontal fracture through the right patella, with proximal displacement of the superior fragment (Figure 1). The CT scan subsequently demonstrated the small amount of comminution associated with this fracture.

This patient was discussed at the local trauma meeting, and given her age, comorbidities, and level of function, the decision to fix these fractures was unanimously agreed. Literature searches were performed at this point to consider the evidence for best fixation. However, we were unable to find any such case presented previously. As such, we sought local expert advice for fixation, based upon the Arbeitsgemeinschaft für Osteosynthesefragen (AO) principles of fracture fixation [2], and planned open reduction and internal fixation of both patella and proximal tibia with plate and screw fixation. This was undertaken two weeks post-injury, to allow for soft tissue resuscitation and to optimise the patient for anesthesia.

Operative treatment

In isolation, the management strategies for these two injuries would be to approach the patella through a midline incision and the tibial plateau through an anterolateral approach to the proximal tibia. Of course, in executing this plan, there is a strong risk of leaving a narrow skin bridge between the two operative scars. Needless to say, this narrow skin bridge may render that area of skin to necrose with subsequent further complications.

With the above in mind, the decision was made to proceed with a single midline incision to approach both injuries. After passing through the skin, and subcutaneous fat, the dissection proceeds along the lateral gutter, where the iliotibial band (ITB) was identified and split to visualise the split in the fracture tibial surface. The plateau was addressed primarily. The fracture was elevated through the split, supported with Kirschner wire, and filled with small autologous bone chips. Thereafter, with the use of cannulated screws and with a lateral proximal tibial plate, the fracture was lifted and supported, pictured below. A satisfactory hold was achieved with all the screws placed. The ITB was repaired, and following this, our attention focused on the patella fracture.

**Figure 2:**
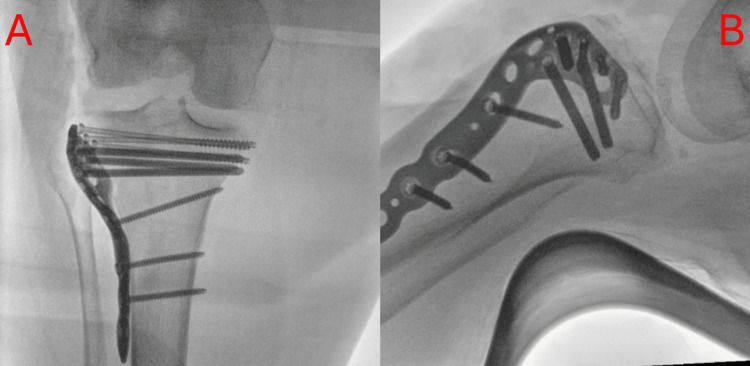
Intraoperative anterior to posterior (a) and lateral (b) X-rays of tibial plateau fixation A) Plain intraoperative anterior to posterior radiograph of the patient's right knee. This image shows lateral plate and screw fixation of the tibial plateau fracture; B) Plain lateral intraoperative radiograph of the patient's right knee. This image shows the lateral plate, with screw fixation, of the tibial plateau fracture.

**Figure 3:**
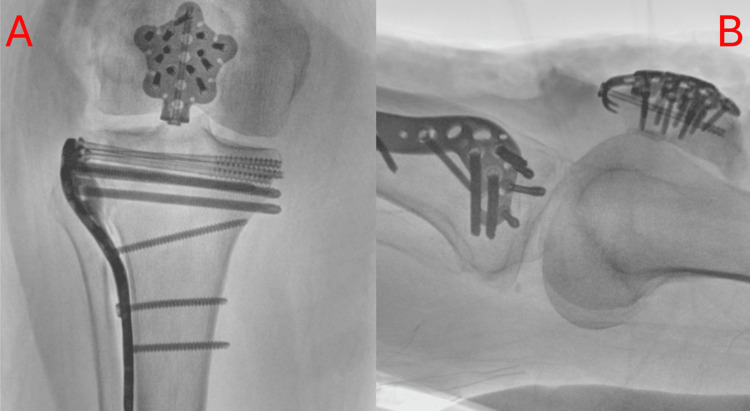
Intraoperative anterior to posterior (a) and lateral (b) X-rays of patellar fixation A) Plain intraoperative anterior to posterior radiograph of the patient's right knee. This image shows the patellar fracture fixed with a plate and screws. It also shows the tibial plateau fracture fixation, seen in Figure 2; B) Plain lateral intraoperative radiograph of the patient's right knee. This image shows the patellar fracture fixed with plate and screws. It also shows the tibial plateau fracture fixation, seen in Figure 2.

To address the patella, using the same incision, we were able to clear the soft tissue and retinaculum superficial to the patella. As proven by the CT, the fractured patella was seen to be communited, distal, and more oblique than initially anticipated. Therefore, the decision was made to proceed with a patella plate (over a tension band technique). The fracture fragments were opposed and head with wires. Following this, the appropriately sized patella plate was utilised. An initial single partially threaded retrograde screw was inserted from the smaller (distal) fragment to the larger (proximal) fragment. The screw was partially threaded to reduce and apply compression at the fracture site. The patella plate, therefore, was used in neutralisation mode to reduce the tension across the fracture site. All screws utilised in securing the patella were locking screws and 2.7mm in diameter, as shown in Figure 3.

The retinaculum was reinforced using a cerclage suture, and following this, the wound site was closed from superficial to deep.

On the table, the patient was placed in a hinge knee brace locked in extension. The patient was not allowed to weight-bear on this leg until the fracture had clinically and radiographically healed. In addition to this, knee flexion was increased by 30 degrees every two weeks, with the plan being to remove the brace after six weeks - after confirmation of a healed patella fracture.

Outcome and follow-up

The patient recovered well on the ward and received an intensive course of physiotherapy. She was placed in a hinged knee brace and was told not to weight bear for eight weeks. The knee brace was initially locked at zero degrees (extension) for two weeks, with 30 degrees being added every two weeks until the brace was unlocked at eight weeks.

**Figure 4:**
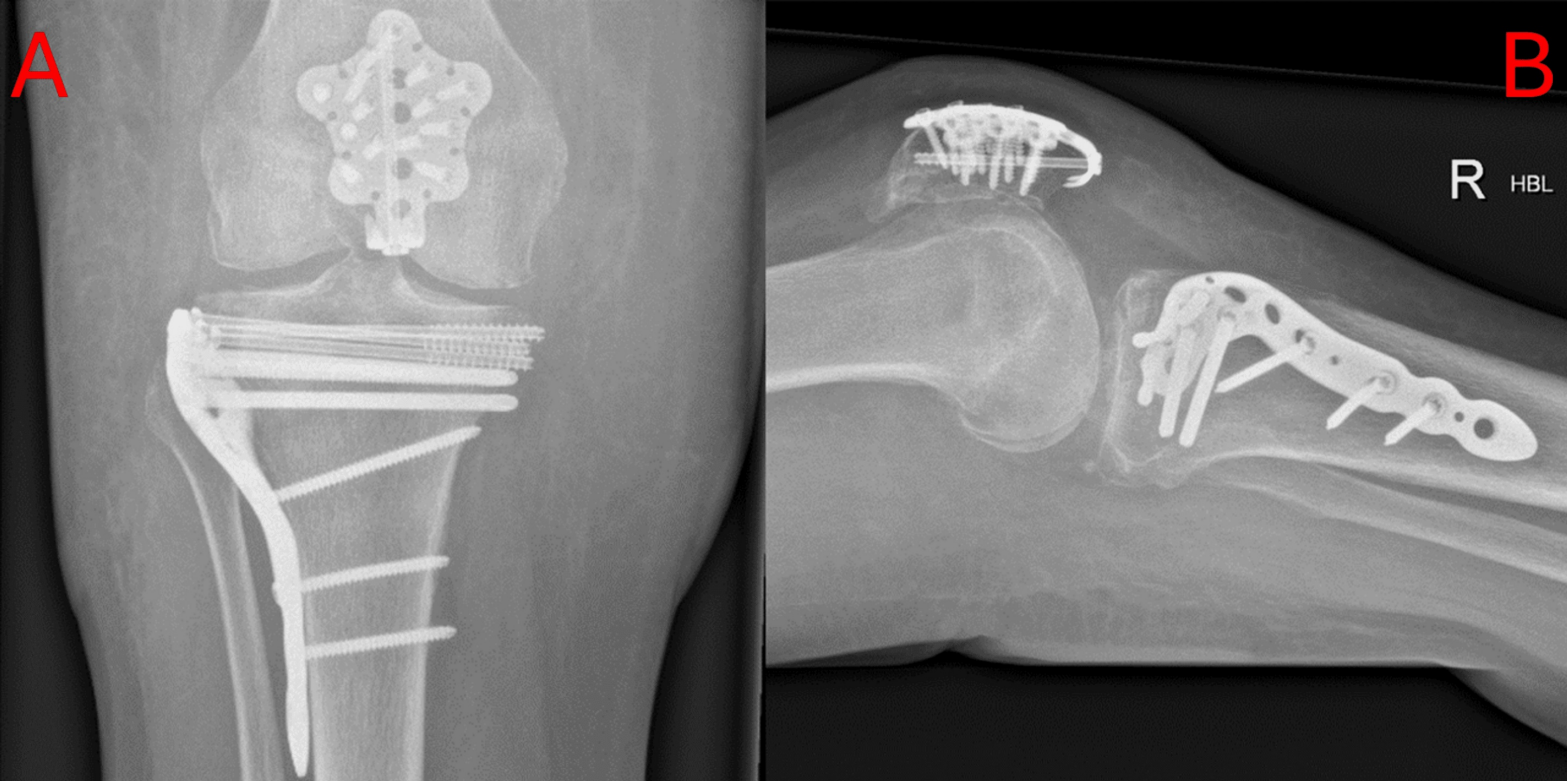
Anterior to posterior (a) and lateral (b) X-rays of right knee three months postoperatively. A) Plain anterior to posterior radiograph of the patient's right knee. This image shows adequate fixation of both the patella and the tibial plateau fracture at three months after the operation; B) Plain lateral radiograph of the patient's right knee. This image shows adequate fixation of both the patella and the tibial plateau fracture at three months after the operation.

She was compliant with physiotherapy and discharged home with two weekly outpatient follow-ups. There have been no significant postoperative complications as of three months post-op. Her three-month x-rays, included below in Figure 4, show that the fracture remains well reduced and is healing. The fixation is stable with no migration of the hardware.

**Figure 5:**
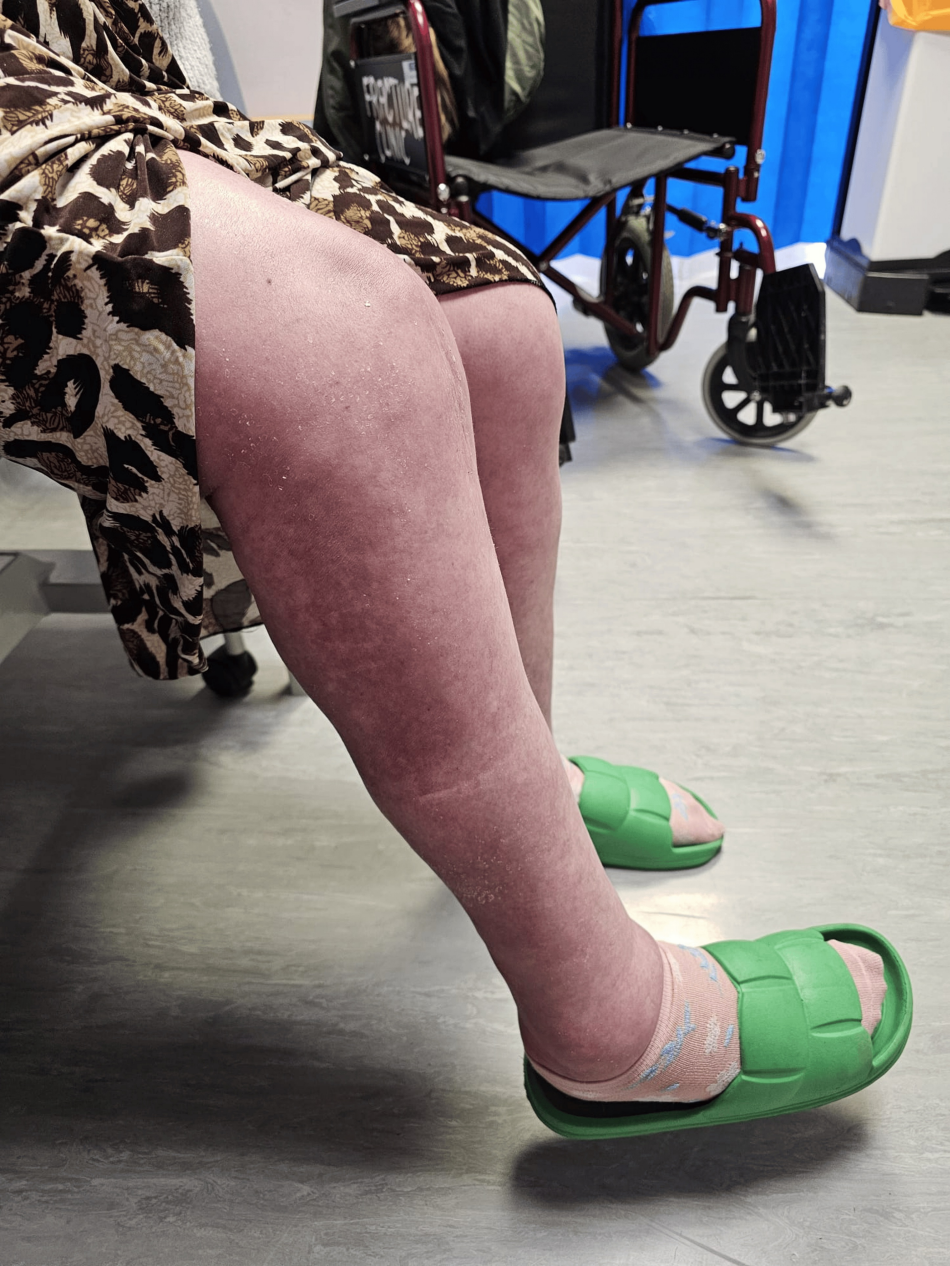
Patient's knee in flexion three months after the operation

**Figure 6:**
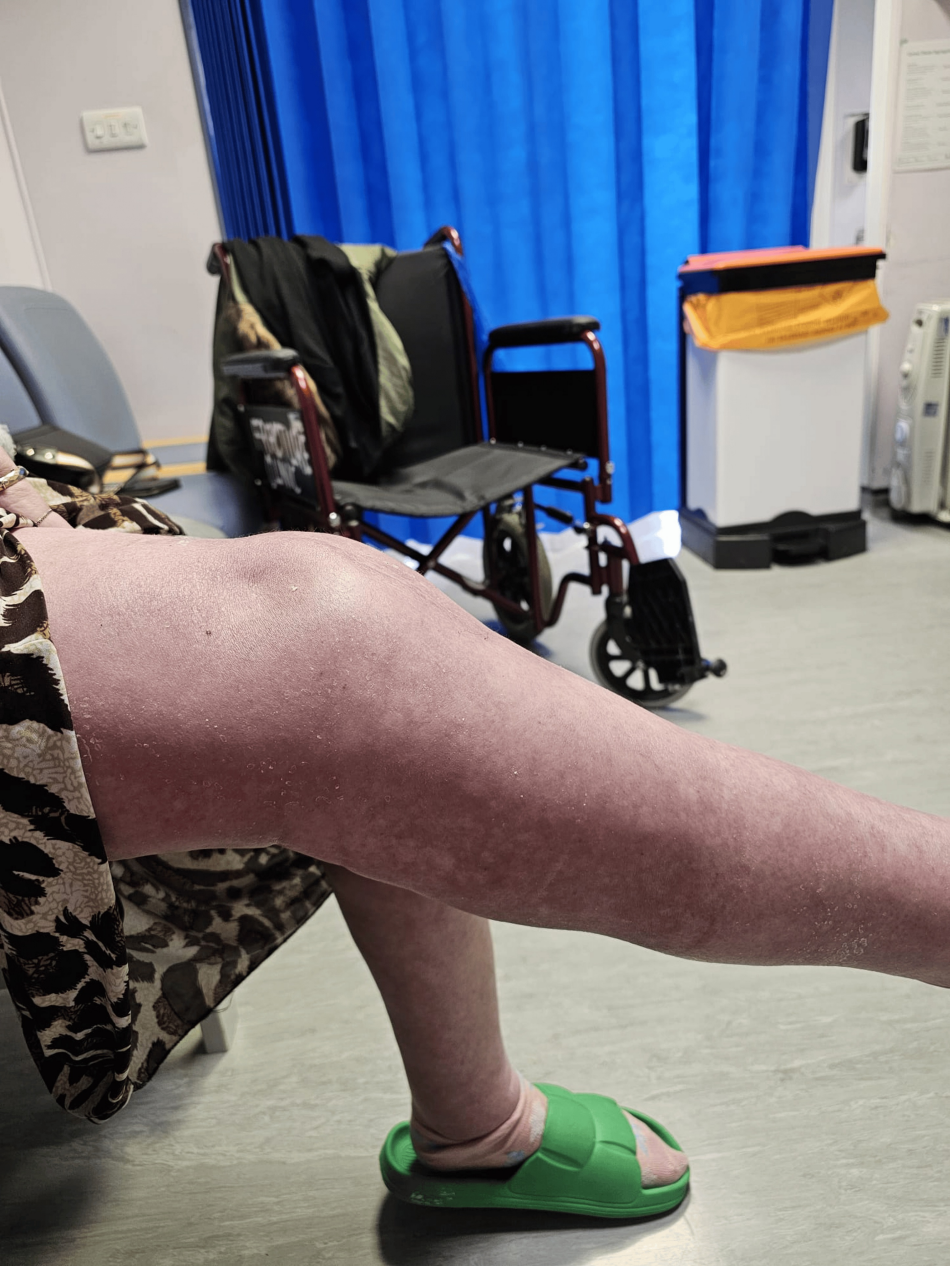
Patient's knee in extension three months after the operation

Figures 5 and 6 show the patient's knee in flexion and extension at three months follow-up. There was a 10-degree extension deficit, and the patient could flex to 60 degrees.

**Figure 7:**
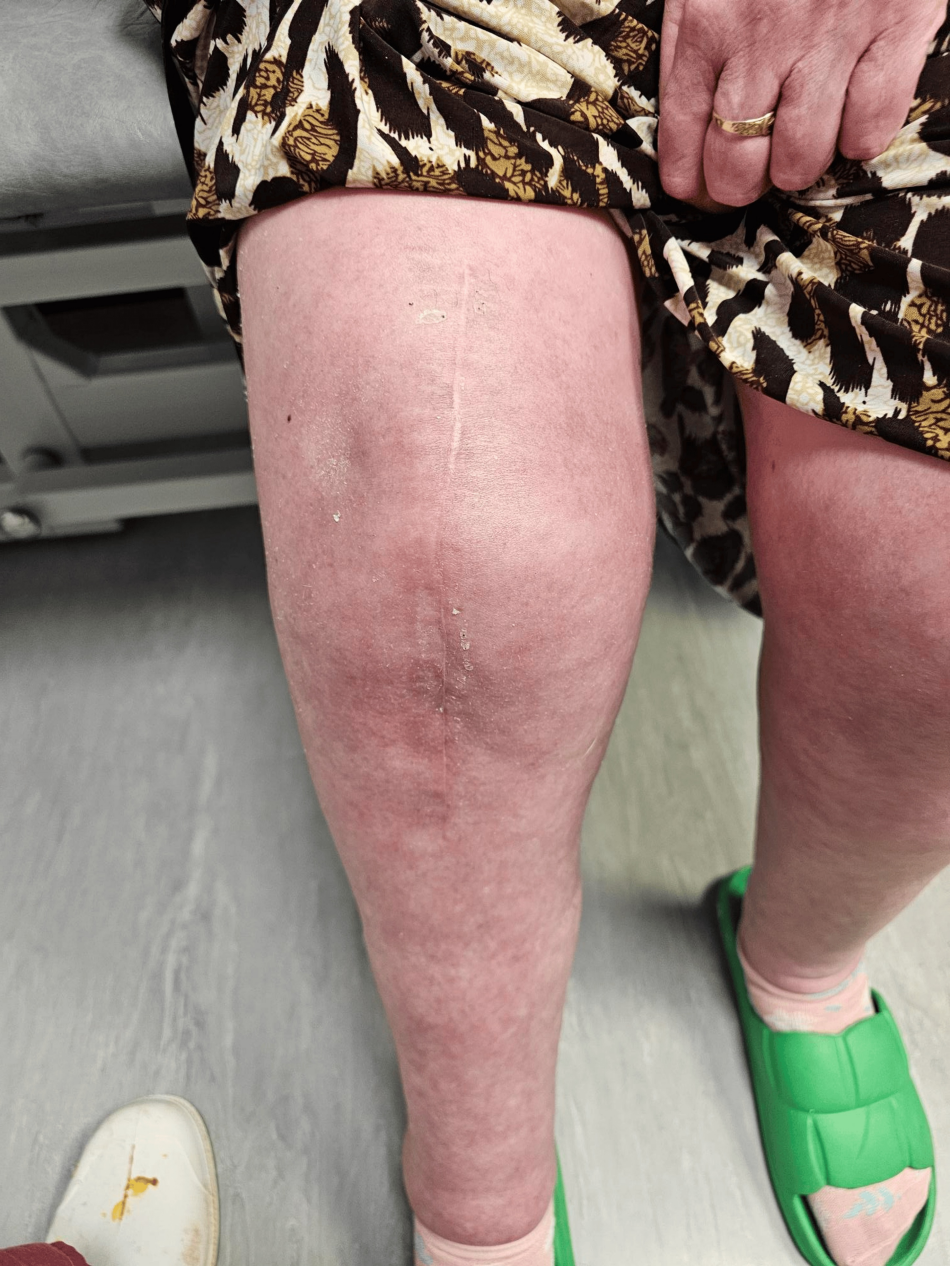
Satisfactory scar healing at three months postoperatively Picture of the single midline scar at three months postoperatively, well healed.

Her scar (Figure 7) showed satisfactory healing, with the patient observing an area of numbness overlying the wound. This is an expected consequence and could not be avoided.

She has been mobilising full weight bearing. While her progress is remarkable, it has been hindered somewhat by communication difficulties with the community physiotherapy service. This has since been rectified.

## Discussion

We consider this case important for presentation due to the unique injury pattern, the importance of anatomical fixation in fractures extending into a joint's articular surface, and our novel approach. As there was no existing operative framework for these injuries that we could base our treatment upon, we relied on the principles of fixation.

Principles of tibial plateau fixation

Fractures that involve the joint surfaces require anatomical fixation [3], avoiding 'steps', which implies a difference in the height of the intraarticular surface of the tibial aspect of the knee. Tibial plateau fractures are, by definition, intra-articular fractures. Therefore, this fracture pattern requires careful reduction to anatomical position and robust fixation to avoid complications, such as osteoarthritis.

Additionally, effective care for the soft tissues is needed peri-operatively. In high-impact injuries, such as tibial plateau fractures, there is significant soft tissue swelling. This can lead to complications intraoperatively, as appropriate coverage of metalwork cannot be achieved. As such, elevation of the limb above the level of the heart is vital. As no operation is possible in this early phase, it is important to splint the injury. We managed the patient's leg in a knee immobilising splint to allow elevation and reduce pain in the acute phase.

Finally, safe and early mobilisation of the joint postoperatively is required to avoid stiffness of the joint and additional medical complications, such as venous thromboembolism (VTE). This, again, was challenging in this case, as the overlying patellar fracture limited our options to allow flexion as the knee. We chose a hinged knee brace, as its use in the management of patellar fractures is commonplace [4] to provide effective support of the knee joint. Initially, the joint was locked in extension to allow healing; however, early motion was allowed at the first follow-up to avoid complications. Additionally, the patient was provided with VTE prophylaxis in the form of low molecular weight heparin, with education on administration. Non-chemical VTE prophylaxis was also used.

Principles of patellar fracture fixation

The patellar is the largest sesamoid bone in the body and serves an important function in the extensor mechanism of the knee [4]. The mechanism of injury determines the fracture pattern. Common mechanisms of patellar fracture are either three-point bending or direct blows [5].

Restoring the extensor mechanism of the knee is the main goal for patellar fracture fixation. Simple, transverse fractures of the patellar fracture may be managed with tension band wiring, in which stainless steel wires are wrapped around two Kirschner wires to convert tension forces at the fracture site into compressive forces to facilitate direct bone healing. However, in this case, due to the highly comminuted nature of the fracture, this was not a suitable option. Instead, we undertook fixation with a tension band plate to achieve a similar compressive effect across all of the significant fragments.

Similar to tibial plateau fractures, complications of stiffness and VTEs must be considered. Patient compliance and education with respect to physiotherapy is an important consideration for successful outcomes, as it is one of the leading causes of fixation failure [6]. Additionally, due to the superficial nature of the patella, symptomatic hardware is also a reported complication [4]. A recent single-centre analysis has found one in five (22/106) patients needed a second operation to remove metalwork [7]. In our follow-up of this patient thus far, we do not report any such complication; however, we will ensure through follow-up and consider removal of hardware, should this occur.

## Conclusions

Ipsilateral patella and tibial plateau fractures are rare, high-impact, unstable fractures. They are associated with a multitude of complications, including arthritis, non or malunion, and medical complications such as deep vein thrombosis. Fixation of these injuries may be indicated, as it was in this case, given the patient's good premorbid baseline. This decision, however, should not be taken lightly, and input from the full trauma multidisciplinary team should be sought to ensure the best outcomes. Approaching these injuries through two incisions presents challenges for soft tissue healing; therefore, using a single midline approach may be considered and is viable based on our experience. Fixation through this midline incision is technically challenging. However, it is a suitable option for returning the patient's long-term knee function. This approach has thus far presented no complications, save for an area of numbness overlying the wound, an expected complication for which the patient was counselled preoperatively. Postoperatively, rigorous physiotherapy and follow-up are needed in these cases to ensure adequate and safe early mobilisation. Patient education and compliance are also important, and were facilitated with clear postoperative instructions and family counselling.

In conclusion, we hope our experiences with this rare injury pattern and our chosen method of fixation may serve as a framework for the management of similar injuries in the future and that this case report may contribute to the literature where we could find no other examples of management of such an injury pattern.
